# Applying the reference values of real-ear-to-coupler-difference for deaf and hard-of-hearing children in Taiwan: Cautions and considerations

**DOI:** 10.1371/journal.pone.0295236

**Published:** 2023-12-01

**Authors:** Chun-Yi Lin, Yi-ping Chang, Ying-Chuan Julie Ma

**Affiliations:** 1 Department of Audiology and Speech-Language Pathology, Asia University, Taichung, Taiwan; 2 Speech and Hearing Research Institute, Children’s Hearing Foundation, Taipei, Taiwan; 3 Department of Audiology and Speech Language Pathology, Mackay Medical College, New Taipei City, Taiwan; 4 Audiology Department, Children’s Hearing Foundation, Taipei, Taiwan; Universiti Malaya Fakulti Perubatan: University of Malaya Faculty of Medicine, MALAYSIA

## Abstract

Measurement of real-ear-to-coupler differentials (RECDs) is a critical part of the hearing aid (HA) verification process. This study examines the validity of reference RECD values preset by the HA analyzer, Audioscan RM500, for deaf-and-hard-of-hearing (DHH) children in Taiwan. RECD measurements were performed on 658 ears of DHH children. A linear mixed model was used to analyze the reference and measured RECD values. The findings revealed slight disparities between normative RECD values from North America and those observed in Taiwanese DHH children. While generally small (less than 5 dB), these differences imply potential challenges in achieving optimal HA fitting in specific scenarios. Therefore, we recommend individualized RECD/REM measurements for cases of poor auditory performance, certain frequency ranges, or notable variations in ear canal volume. From a clinical perspective, while broadly applicable, the use of North American RECD normative data in Taiwan requires cautious consideration of potential minor variations. This study contributes to current knowledge by affirming the use of a Western RECD database for Taiwanese DHH children. However, we underscore the ongoing importance of individualized HA fitting strategies, particularly for cases with stagnant intervention progress. While built-in RECD reference values can offer preliminary fitting guidance, especially in busy clinical settings, our study sheds light on the circumstances where caution is essential. Audiologists can efficiently allocate their time and effort by focusing on personalized RECD measurements for cases exhibiting suboptimal intervention outcomes, thereby effectively optimizing HA gain settings.

## Introduction

In Taiwan, with the inception of universal newborn hearing screening in March 2012, infants with hearing loss can be identified at birth and enrolled in intervention programs earlier than before. For these children whose parents choose spoken language as the primary communication mode, optimal amplification fitting provides a crucial foundation for auditory development. However, young children have a limited ability to express whether the amplification is sufficient and appropriate. Consequently, pediatric audiologists mainly rely on objective validation and verification for hearing aid (HA) fitting. Objective HA validation refers to speech audiometry. However, the limited cognitive and language abilities as well as limited concentration time of young children may compromise the reliability of the speech detection tests. As a result, audiologists use objective HA verification to evaluate the benefits of HAs without having the child respond.

HA verification can be completed via a simulated real ear measurement (SREM) or on-ear real ear measurement (REM). While administering SREM, audiologists connect the HA to the coupler in the test box and input hearing thresholds of the patient into the HA analyzer program, which calculates the target gain. Audiologists examine whether this gain matches the prescribed target gain. In contrast, on-ear REM is performed by inserting a probe microphone in the ear canal of a patient, to identify the sound pressure level (SPL) of an amplified signal in the ear canal and obtain it at the eardrum. Real-ear-to-coupler-difference (RECD) is a REM measure that considers the ear canal characteristics of a particular person and is used in conjunction with simulated real-ear verification to verify whether the HAs are fitted appropriately in children. After obtaining the RECD values acquired in a short period of time, audiologists can apply them in the following verification process without having to keep the child seated.

Turning our focus to the methodological aspects of HA verification, it’s noteworthy that according to the American Academy of Audiology [1], the most effective method for verifying hearing aid fitting involves the use of a probe-microphone system to ensure that hearing aid output levels match the prescribed targets. Achieving appropriate output levels align with these prescriptive targets has been demonstrated to yield substantial benefits, including enhancing aided audibility [2], speech perception [3], and language development [4]. Moreover, the impact of not adhering to these prescriptive targets extends beyond mere fitting deviations. Research indicates that children whose hearing aid outputs closely match targets for audibility across different inputs and frequencies exhibit superior speech recognition outcomes compared to peers with suboptimal fittings [5]. This finding underscores the repercussions of failing to meet prescriptive targets, affecting metrics such as maximum power output (MPO) and input levels spanning from soft to loud. In addition, it has been shown that inadequate MPO levels falling below the target can limit aided speech recognition in noise [6]. Furthermore, children with higher root mean square errors for speech input levels experience reduced aided audibility and poorer word recognition performance in quiet. The misalignment in specific frequency bands also negatively influences outcomes. Notably, the presence of high-frequency hearing loss in children adversely impacts the sensation level of speech at 4 kHz, resulting in worsened aided speech recognition in both quiet and noisy environments.

While average RECDs have been reported for Western population [7,8], there are limited data on RECD measurements for Eastern population [9]. In particular, there is no clinically available database of reference RECDs for Taiwanese children. Although Ma and McPherson [10] investigated RECD data for the Chinese population, the study included young adults having normal hearing instead of the pediatric population. The main purpose of RECD measurement is to verify the HA setting for DHH children. However, the reference RECDs preset in HA analyzers (e.g., Interacoustic and Audioscan) are derived based on the data for normal hearing children from the Western countries. Although individually measured RECD has been emphasized, during busy clinical situations, audiologists in Taiwan usually rely on reference RECDs. Moreover, when conducting tympanometry, audiologists at Children’s Hearing Foundation (CHF) noticed that the ear canal volume of DHH children in Taiwan seems different from Western children. Considering that the size of the ear canal volume also influences the sound pressure in the ear canal, it is important to examine the applicability of the reference RECDs while fitting HAs for children with DHH in Taiwan.

This study aimed to investigate the measured RECDs of DHH children in Taiwan and the applicability of the reference RECDs preset in the verification equipment. The data were visualized during an initial examination. A linear mixed model was constructed to determine the presence of differences between the measured and reference RECDs. In addition, the study delves into clinical implications derived from the statistical analyses.

## Methods

### Participants

The RECD values of 671 ears were collected from the audiological records of children aged <16 years who underwent audiological assessment at the CHF. The data were collected only from children whose parents/guardians gave their written consent to use their data for research purposes.

Due to the fact that the RECD measurements were part of the regular audiometric evaluation process, virtually no risk was involved for the participants. In addition, the researchers are unable to identify individual patients through any linkage file or code since the data had already been de-identified by the time they retrieved it. Thus, this study does not qualify as human subject research and does not require IRB approval. Data collected included age in months, middle ear function, RECD values, and assessment/measurement reliability.

The data were excluded under the following circumstances: (1) the child had any middle ear disorders, (2) any situation that may cause unreliable measurements; for example, the child was restless during testing, the probe tube was not placed properly within 5 mm of the eardrum, or the ear tip was not inserted fully into the ear canal. After the exclusion criteria were applied, the RECD values of 658 ears were analyzed. The age of the children at testing ranged from 6–172 months, and the mean age was 52.7 months.

### Procedure

RECD data were collected from the routine hearing test procedure, RECD values were measured as a part of the HA verification (the test procedure also included otoscopy, tympanometry, and audiometry). Otoscopy (Welch Allyn Halogen HPX™, Welch Allyn Inc., Skaneateles Falls, NY, USA) was first performed to confirm the absence of cerumen/debris or other outer ear disorders. Then, tympanograms were generated using GSI TympStar™ (Grason-Stadler, Eden Prairie, MN, USA) to assess middle ear function, as instructed by Gelfand [11]. Hearing thresholds were obtained using behavioral audiometry using GSI 61 (Grason-Stadler, Eden Prairie, MN, USA) according to the developmental age of the child [11].

HA was verified in a quiet room using Audioscan (RM500 Version 3.24, Etymonic Design Inc., Ontario N0L 1G0, Canada) which is still widely used in Taiwan despite being discontinued. Before the RECD measurement, the standard 2-cc HA-2 coupler and microphone were calibrated according to the Audioscan RM500SL manual [12]. The same foam tip was used throughout the measurement procedure, as variations in domes or tips could potentially influence the ear canal volume [13]. The probe tube was connected to the probe microphone, and the E-A-RLINK™ pediatric foam tip was connected to the RE770 transducer, which transferred the frequency stimuli and received the resonance sound reflected from the ear canal. The probe microphone and transducer were connected to RM500, which assessed the RECD values. The participants sat approximately 30**−**50 cm in front of the RM500. The probe tube length (foam tip length: 12.7 mm, the probe tube: 3**−**5 mm from the foam tip) was measured. An otoscope was used to inspect that the insertion depth of the probe tube was within 5 mm of the eardrum. After ensuring the distance of the probe tube and stabilizing it, the foam tip was fully inserted into the ear canal without changing the probe tube position. Following the setup of the probe tube and foam tip, RECD measurements were completed.

### Analyses

Data visualization. The reference dataset was constructed by extracting the reference RECD values preset in the RM500, to compare with the measured RECD data. These age-related reference RECDs were obtained using foam-tip measurements (in accordance with ANSI S3.46 methods: RECD On-ear measurement) coupled with the Audioscan RECD transducer and a 2-cc HA-2 coupler, as set in the RM500 instrument [12]. Each measured RECD value for a specific frequency was compared with the age-matched reference RECD value for the same frequency from the reference database; for example, the measured RECDs of both the left and right ears of a 6-month-old infant for a frequency of 500 Hz were compared with the 6-month reference RECDs from the built-in database of the RM500 (Table 1).

**Table 1:** Illustration of data construction for statistical analyses.

Source		Measured RECD									Reference RECD								
Participant (age in months)		250	500	750	1 k	1.5 k	2 k	3 k	4 k	6 k	250	500	750	1 k	1.5 k	2 k	3 k	4 k	6 k
1 (57)	L	7	9	10	10	12	13	13	15	16	3	5	7	10	10	12	11	15	19
	R	9	11	11	11	12	14	14	15	15	3	5	7	10	10	12	11	15	19
2 (38)	L	9	11	11	11	13	12	14	10	19	3	5	7	10	11	13	11	15	19
	R	7	9	9	8	10	15	13	5	7	3	5	7	10	11	13	11	15	19
…	…	…	…	…	…	…	…	…	…	…	…	…	…	…	…	…	…	…	…

L, left; R, right; Freq, frequency; Measured RECD, the value measured from children with DHH; Reference RECD, the value preset in the equipment.

Note: The gray part of the table was constructed using the RM500 reference database. For each measured RECD value, a reference RECD value, matched with age and frequency, was used in the dataset. Note that for both the left and right ears of the same participant, the matched reference values were the same.

The average measured and reference RECDs were plotted on a line chart as a function of frequency to investigate the relationship between the RECD and frequency, and to evaluate whether that relationship was different for the measured and reference datasets. In addition, the data points across ages (0–192 months) were plotted on a scatter plot for each frequency from 0.25 kHz to 6 kHz.

Linear mixed model. The measured RECDs were from both the left and right ears of each participant; therefore, the dataset contained highly correlated data. Consequently, a linear mixed model was used to resolve the dependency issue. Table 2 lists the fixed and random effects considered in the model. The fixed effects included the source of RECDs (measured or reference), chronological age, ear (left or right), and frequency. Frequency is treated as a continuous and logarithmic variable due to its representation of a crucial physical property of sound. The logarithmic scaling choice is in accordance with the non-linear response of the human auditory system to sound stimuli. This approach facilitates the effective capture of variations in RECD values across different frequency levels while preserving the underlying trend observed in audiological measurements.

**Table 2:** Fixed and random effects in the linear mixed models.

Effects	Fixed/Random	Type	Note
Source	Fixed	Categorical	2 levels: 0 = reference; 1 = measured
Age	Fixed	Categorical	6 levels with an approximately equal number of participants in each age group: 0 = 5−23 months 1 = 24−35 months 2 = 36−47 months 3 = 48−56 months 4 = 57−81 months 5 = 82−172 months
Ear	Fixed	Categorical	2 levels: R = right; L = left
Frequency	Fixed	Continuous	logarithm value with base 2
Participant	Random	Categorical	

In addition, two interaction terms were also included: (1) the interaction between the RECD value source and frequency, and (2) the interaction between the RECD value source and age. These two interaction terms were included to explore whether the effect of age or frequency on RECDs was different between the Western reference database and the measured Taiwanese data. As mentioned above, a mixed model was used to account for the correlation issue. Specifically, the RECDs measured from the left and right ears of a particular participant were highly correlated. Therefore, the random part of the model incorporated a random term for the participant, including the slopes for the ear. The linear mixed model was generated using the mixed() function in the R package afex [14] and can be expressed as “RECD ~ source + ear + age + frequency + source:frequency + source:age + (ear | participant)”.

## Results

### Data visualization

**Fig 1:**
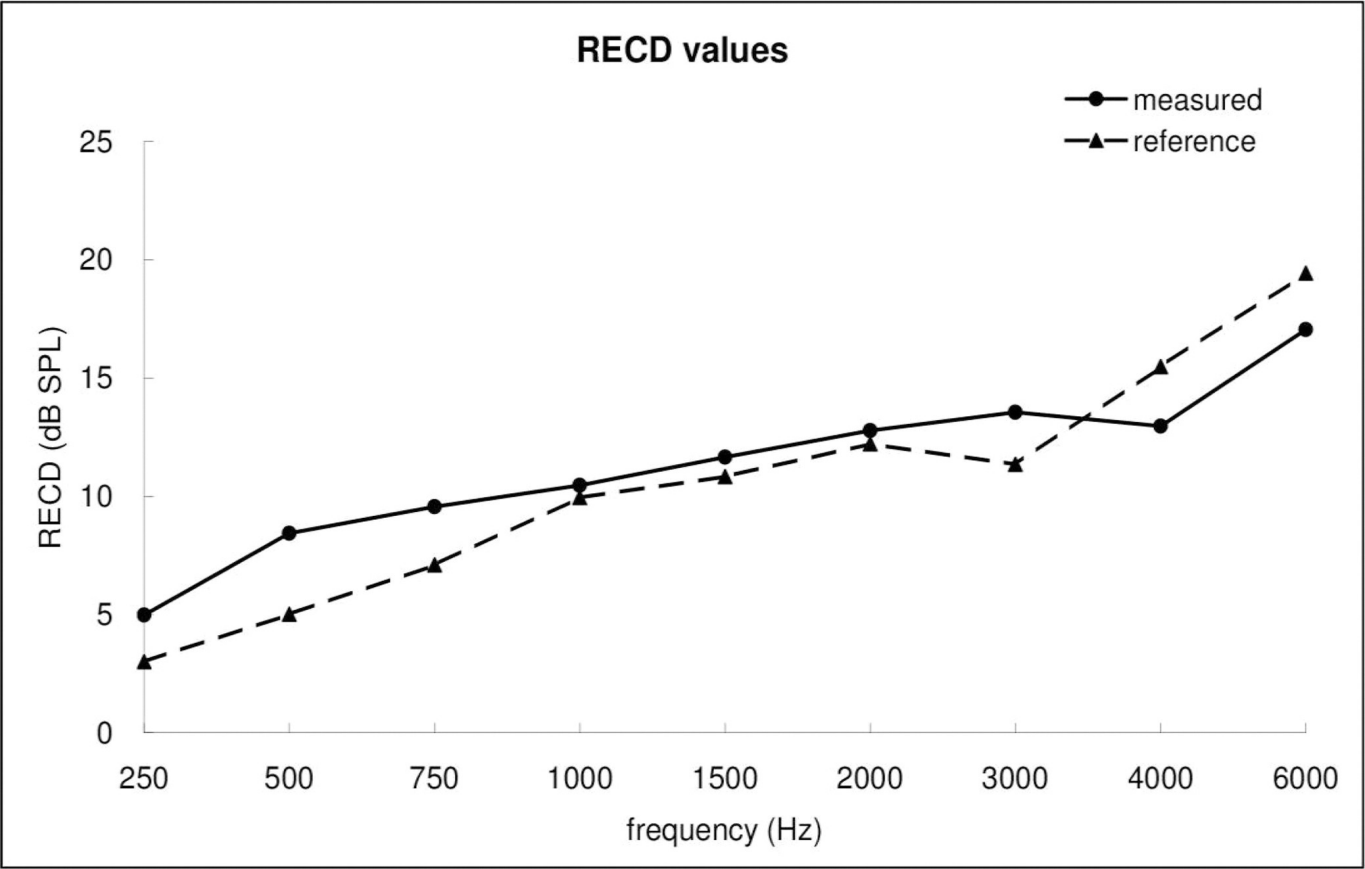
Comparison between reference and measured RECD values across the frequency range.

Fig 1 displays the mean RECDs measured in this study and the reference RECDs preset in RM500 as a function of frequency. An increasing trend from low to high frequencies was observed for both the measured and reference RECDs. Visual observation revealed a possible interaction between the source (measured vs. reference) and frequency. For lower frequencies (0.25–3 kHz), the measured RECDs were greater than the reference values in RM500, whereas for higher frequencies (4 kHz and 6 kHz), the measured RECDs were smaller than the reference values.

**Fig 2:**
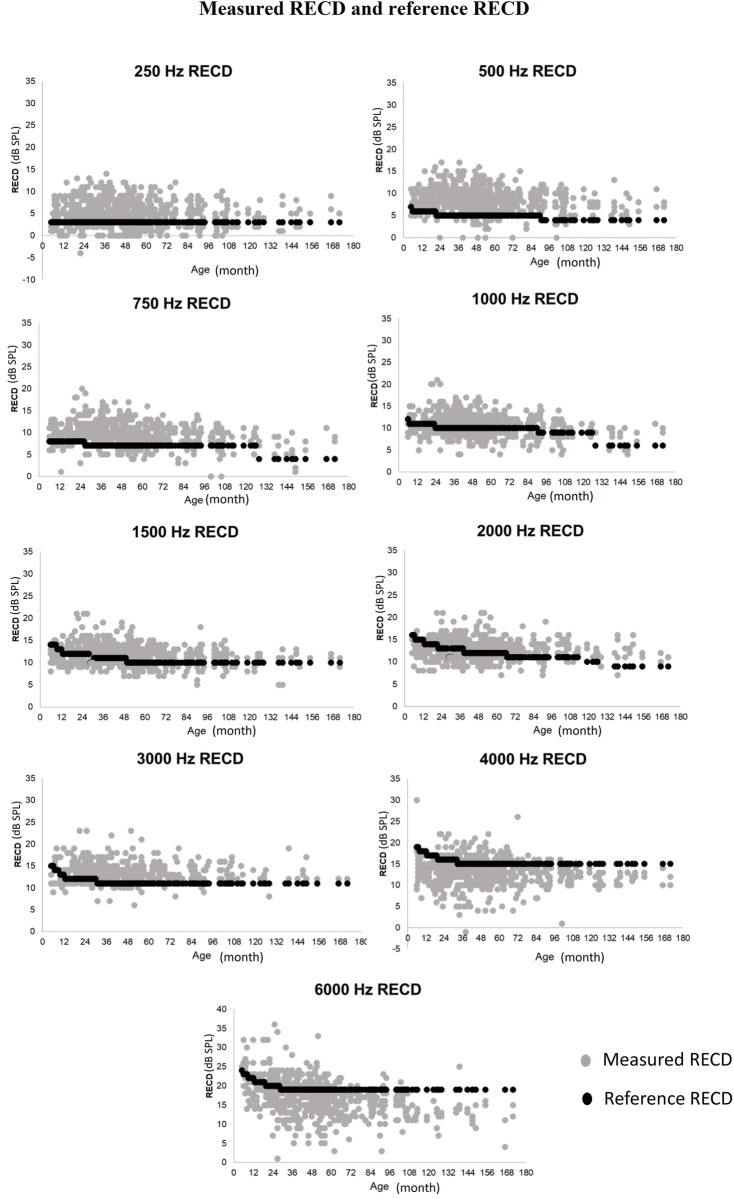
Scatter plots of measured (grey) and reference (black) RECD values across age for each measured frequency.

All data from each ear are plotted in Fig 2, with each data point (grey dot) representing the RECDs measured for the corresponding frequency from each ear as a function of the child’s age. The reference RECDs (black dots) are shown for comparison. The results indicated that the RECD values were highly variable across the participants.

### Linear mixed model

The results of the linear mixed model are presented in Table 3. A significant effect of the source was observed (p < 0.0001), indicating potential differences between the measured and reference RECD values. The estimated difference between the measured and reference RECD values was formulated as follows: -1.07 * log2(freq.) + 12.2 + age_effect, where “age_effect” corresponds to an array of age-specific adjustments: [0, 0.383, 0.233, -0.551, -0.529, -1.03] for age groups [0, 1, 2, 3, 4, 5] (all the numbers are based on the “estimate” in Table 3). For example, the estimated difference for a frequency of 1000 Hz and for the age group 4 (57**−**81 months) between the measured and reference RECD values was, on average, 1.008 dB (-1.07*log2(1000) + 12.2–0.529). Table 4 lists the estimated differences between the measured and reference RECD values according to the frequency and age groups. When the frequency (in Hz) doubled (i.e., the logarithm value of the base 2 increased by 1), the RECD difference decreased by 1.07 dB. The largest discrepancy of 4.06 dB, on average, between the measured and reference RECD values was observed for the age group of 24**−**35 months at 250 Hz. However, this largest discrepancy was clinically marginal as it fell within the test-retest reliability range for probe microphone measures and within a single step (5 dB) on audiogram. As shown in Table 4, the measured RECD values were greater than the reference values in RM500 for lower frequencies, whereas the measured RECD values were smaller than the reference values for higher frequencies. The results are consistent with the observed significant interaction between the source and frequency, as well as with the trend shown in Fig 1. Additionally, for the mid-frequency range (1.5 kHz**−**3 kHz), the discrepancy between the measured and reference RECD values was relatively small. Moreover, the differences between the measured and reference RECD values were relatively large for frequencies <1 kHz and >3 kHz across the age groups, but they were larger for the younger (< 36 months) and older (> 48 months) age groups, respectively. Given the high variability of RECDs among individuals, using reference values may not be optimal for all cases, for example, in specific age groups (< 36 months and > 48 months) and frequencies (<1 kHz and >3 kHz). Therefore, our data provide valuable information for audiologists as references, guiding them in considering individualized approaches in specific scenarios. However, despite these considerations, the observed differences, while statistically significant, fall within the test-retest reliability range, suggesting that the reference values can generally be applied to Taiwanese children, supporting the notion of broader applicability.

**Table 3:** Results of the linear mixed models.

Fixed effects	Estimate	Standard error	t value	Pr (>|t|)	Sig.
(Intercept)	-23.3	0.252	-92.402	<0.0001	***
source1	12.2	0.308	39.658	<0.0001	***
earL	-0.199	0.045	-4.416	<0.0001	***
age1	-0.622	0.200	-3.109	0.0020	**
age2	-0.703	0.202	-3.484	0.0005	***
age3	-0.771	0.196	-3.937	<0.0001	***
age4	-0.851	0.201	-4.236	<0.0001	***
age5	-1.05	0.201	-5.216	<0.0001	***
frequency	3.31	0.020	167.209	<0.0001	***
source1: frequency	-1.07	0.028	-38.115	<0.0001	***
source1: age1	0.383	0.136	2.808	0.0050	**
source1: age2	0.233	0.138	1.694	0.0902	N.S.
source1: age3	-0.551	0.133	-4.126	<0.0001	***
source1: age4	-0.529	0.137	-3.863	0.0001	***
source1: age5	-1.03	0.137	-7.524	<0.0001	***

Significance codes

*** <0.001

** <0.01

* <0.05; Sig., significant; N.S., not significant; Pr, probability.

**Table 4:** Estimated differences between measured and reference RECD values according to frequency and age groups.

Freq. (Hz)	log_2_ (freq.)	age group:0 5−23 mo.	age group:1 24−35 mo.	age group:2 36−47 mo.	age group:3 48−56 mo.	age group:4 57−81 mo.	age group:5 82−172 mo.
Compared with age group: 0		-	+0.383	+0.233	-0.551	-0.529	-1.03
250	7.966	3.677	4.060	3.910	3.126	3.148	2.647
500	8.966	2.607	2.990	2.840	2.056	2.078	1.577
750	9.551	1.981	2.364	2.214	1.430	1.452	0.951
1000	9.966	1.537	1.920	1.770	0.986	1.008	0.507
1500	10.551	0.911	1.294	1.144	0.360	0.382	-0.119
2000	10.966	0.467	0.850	0.700	-0.084	-0.062	-0.563
3000	11.551	-0.159	0.224	0.074	-0.710	-0.688	-1.189
4000	11.966	-0.603	-0.220	-0.370	-1.154	-1.132	-1.633
6000	12.551	-1.229	-0.846	-0.996	-1.780	-1.758	-2.259

Freq., frequency; mo., months.

Note: A positive difference indicates a greater value for the measured RECD value than the reference value. Non-significant estimate was observed for age group 2, as indicated by the gray shadowed cells in the table.

A positive coefficient for the fixed effect of frequency indicated that the RECD values increased at higher frequencies. Specifically, the estimated RECD value increased by 3.31 dB when the frequency was doubled in Hz. This is consistent with the trend observed in Fig 1. In addition, the coefficients for all age groups were negative and decreased with age, implying that the RECD values decreased with age. This result is consistent with the trend shown in Fig 2.

## Discussion

To verify HAs in Taiwanese children with hearing loss, reference RECD values based on Western populations are often utilized due to the absence of Eastern-based reference databases. This study aimed to assess the applicability of these Western-based reference values to Taiwanese children. The analysis demonstrated a significant difference between the measured RECDs for Taiwanese children and those from the RM500 database. The results revealed that the overall differences were relatively small, suggesting the use of normative data could be viable for most children. However, our investigation revealed a statistically significant variation. Despite this statistical significance, it’s crucial to recognize that these differences fall within the test-retest reliability range, indicating a level of consistency. Nonetheless, this finding underscores the reality that even adherence to protocol-guided RECD measurements can still yield noticeable variations, particularly at low and high frequencies, as opposed to mid-frequencies. These nuanced differences highlight the paramount importance of fitting hearing aids to precise target values, especially considering the substantial inter-subject variation in children. Caution is advised when applying RM500 reference RECDs for Taiwanese children, particularly since low and high frequencies contribute significantly to Mandarin speech comprehension. This cautionary approach is particularly relevant for children with compromised auditory performance or stagnant habilitation progress, where individualized RECD measurements could yield more tailored outcomes.

While acknowledging the general applicability of reference RECDs for initial fitting in Taiwanese DHH children, our study provides valuable insights for audiologists. Further delving into the specifics of our findings, the measured RECDs for low frequencies (0.25–3 kHz) were found to be greater than the reference values. This stands in contrast to the outcomes for higher frequencies (3–6 kHz). Martin, Westwood and Bamford [15] reported increased RECDs in children during episodes of otitis media with effusion (OME). However, we included only participants with normal middle ear function. Therefore, the larger RECDs at lower frequencies were unlikely to be caused by OME. Sachs and Burkhard [16] showed that there is a radiation effect when a sound wave travels from the earmold tube to the outer ear canal, which causes the energy to disperse. They suggested that the probe tube tip should extend by 3**−**5 mm from the earmold sound bore. Our measurements followed this procedure; however, the measured RECDs for frequencies of 0.25 kHz**−**3 kHz were still significantly larger than the reference values. In addition, higher RECDs at low frequencies were also reported by Liu and Lin [17]. Although they reported that the RECDs between adults and children were not significantly different, the RECDs were greater for children at all frequencies. Moreover, Liu and Hsu [9] compared the Chinese and Western population studied by Feigin et al. [18] and the results indicated smaller RECDs for the Chinese population than for the Western population at frequencies of 3 kHz**−**6 kHz. This result is consistent with the results of our study, indicating that RECDs may differ slightly among populations.

The measured RECDs were greater at low frequencies and smaller at high frequencies, compared with the reference values. This result may be caused by the differences in the geometry of the ear canal between the Western and Eastern children. The ear canal is essentially a tube that is open at one end and closed at the other, acting as a resonating body. Generally, the natural resonance frequency of the ear canal is four times its length. It is calculated using the following formula: f = c/(4L) (f = resonant frequency; c = velocity of sound in air; L = length of ear canal) [19]. The RECD value is equal to the SPL in the ear canal subtracted by the coupler SPL. Due to the fixed geometry of the coupler, the RECD value is mainly determined by the resonant frequency in the ear canal. Compared with the reference values, the results of this study indicated higher measured RECDs at low frequencies and smaller measured RECDs at high frequencies. This could be due to differences in the volume of the ear canals of DHH children from Eastern and Western countries. As previous studies have suggested, individual variations in RECDs can range from ±5.6 dB at 500 Hz to ±10.9 dB at 6000 Hz compared to reference values, and these variations may be influenced by factors such as head circumference and ear canal growth [18,20,21]. In the present study, we observed higher measured RECDs at low frequencies and smaller measured RECDs at high frequencies compared to the reference values, which could be attributed to differences in the ear canal geometry of DHH children from Eastern and Western countries. Specifically, the natural resonance frequency of the ear canal, which is mainly responsible for determining the RECD value, is dependent on factors such as the length of the ear canal and the volume of the ear canal [19].

This assumption was examined by analyzing the ear canal volume of DHH children in the present study and comparing it with previously reported data [22,23,24]. In spite of the fact that the mean ear canal volume of DHH children in Taiwan is similar to that of Western children. It is important to note, however, that Taiwanese children have a greater variance in their ear canal volume than previously documented data across all age groups, particularly for children between the age of 2.8 and 5.8. For Taiwanese children, the 90% range of ear canal volume is 0.86 (1.26–0.4 = 0.86 cc), whereas for Western children, the range is 0.55(0.97–0.42 = 0.55 cc). Taiwanese DHH children have an ear canal volume range that is approximately 41% larger than that of Western children. Since DHH children in Taiwan show large individual differences, it may be more appropriate to measure their individual RECDs during HA fitting rather than using reference values.

Large intersubjective variability was observed for all frequencies in this study (Fig 2). Audiologists may obtain inaccurate target gain if they use reference RECDs rather than measured RECDs during HA verification procedures because the target gain is determined by the hearing threshold and RECD value. Over-amplification may occur if the RECD value is larger than the measured value, and vice versa. High interpersonal variability in RECDs indicates that the poor auditory performance of some children may be caused by the fact that their personal RECDs are significantly different from the reference values. However, in situations where practicality is a concern, preset RECD reference values can provide initial fitting guidance. Our study contributes valuable information for audiologists to discern when to prioritize individualized fitting strategies.

In our study, the reference RECDs at lower frequencies were smaller than the measured RECDs. Therefore, using the reference RECDs to verify HAs for DHH children in Taiwan may potentially cause insufficient target gain for frequencies of 0.25 kHz–3 kHz, especially for younger children (three years old or younger). Furthermore, verifying HAs using reference RECDs may possibly result in over-amplification for frequencies of 4 kHz and 6 kHz, especially for older children (older than four years old), owing to the larger reference RECDs than the measured RECDs. An insufficient or overamplified gain is clinically relevant. For example, tonal languages (such as Mandarin, the primary language spoken in Taiwan) rely on low-frequency information to convey lexical meaning. The acoustic information of tones is mainly contained in lower frequencies, and these tones carry meanings of the words in Mandarin. Insufficient gain at low frequencies can result in difficulties in discriminating tones and distinguishing between words in tonal languages like Mandarin, which may negatively impact a child’s language development. Mandarin features four primary lexical tones (Tone 1: high flat, Tone 2: rising, Tone 3: dipping, and Tone 4: falling) and one neutral tone (Tone 0). Numerical markers are added to syllables to denote the specific lexical tone. For example, /kuai1/ denotes the syllable /kuai/ with a high flat tone (Tone 1), while /kuai4/ denotes the same syllable with a falling tone (Tone 4). In Mandarin, /kuai1/ means “good,” whereas /kuai4/ means “weird.” Consider a scenario where a teacher tells a boy, “You are such a good boy,” but the boy mishears it as, “You are such a weird boy.” Numerous other examples exist in Mandarin, such as (1) /ʂuei3 tɕiau3/ (“dumpling”) and /ʂuei4 tɕiau4/ (“sleeping”); (2) /ʂɭ1 tsɨ0/ (“lion”), /ʂɭ2 tsɨ0/ (“stone”), and /ʂɭ4 tsɨ0/ (“persimmon”); (3) /tʂu1 tsɨ0/ (“bead”), /tʂu2 tsɨ0/ (“bamboo”), and /tʂu4 tsɨ0/ (“pillar”). These examples underscore the significance of tone in Mandarin, as incorrect perception of tones can lead to substantial misunderstandings. Therefore, the potential for insufficient gain at low frequencies due to the application of reference RECDs could affect understanding of speech in Mandarin-speaking children diagnosed with DHH. Acknowledging the overall suitability of reference RECDs for the initial fitting of hearing aids in Taiwanese DHH children, our research offers valuable perspectives for audiologists to spend their time and effort efficiently in clinical settings, particularly in circumstances where individualized RECD/REM measurements for optimal fitting are necessary.

On the other hand, the overamplified gain at high frequencies may also confuse these children when similar consonants are to be discriminated. Moreover, Mandarin comprises numerous high frequency phonemes, characterized by different manners (e.g., stops, affricates, fricatives, retroflexes) and places of articulation (e.g., bilabial, alveolar, palatal, etc.) [25]. It is important to note that these high frequency phonemes play a crucial role in distinguishing meaning in Mandarin. If the overamplified gain impairs the accurate acoustic cues, children with DHH may mishear high frequency minimal pairs, such as /ts^h^ao3 di4/ “grassland” vs. /sao3 di4/ “sweeping the floor,” /tʂu1 tsɨ0/ “bead” vs. /ʂu1 tsɨ0/ “comb,” and /tʂu2 tsɨ0/ “bamboo” vs. /tʂ^h^u2 tsɨ0/ “cook.” This misperception is further compounded by louder aspiration or burst energy, which can blur the acoustic characteristics of speech words. Consequently, children with DHH who perceive tones and consonants incorrectly may encounter significant challenges in effectively learning and communicating within educational environments. These challenges can have an impact on their language development and academic success.

In a previous study, when measured RECDs were used instead of reference RECDs, the hearing threshold was increased by as much as 18 dB [25]. This finding underscores the clinical relevance of accurate RECD measurements in hearing aid fittings for children with DHH. In addition, the authors of the current study encountered a case in which audiological data possibly supported the clinical relevance of even small differences in RECDs. In this case, after adjusting the HA fitting, while the aided thresholds revealed an improvement of only 5 dB, the detection distance of the high frequency consonants /tʰɕ/ and /s/ was significantly prolonged from 2.5 m to 10.5 m, and 9 m, respectively. This finding is particularly relevant in educational settings, as a larger detection distance can provide more incidental learning opportunities for children with DHH, which are crucial for their language development and academic success.

Given the nuanced differences uncovered in this study between measured and reference RECDs, along with variations in ear canal volume between Western and Taiwanese DHH children, it prompts a call for caution among audiologists in Taiwan when incorporating reference RECDs into the HA fitting and verification processes for DHH children. This caution extends globally, emphasizing the need for audiologists worldwide to approach the application of preset reference RECDs with care, particularly when dealing with non-Caucasian children. While acknowledging the practicality of using reference RECDs for initial fitting, our study sheds light on the potential challenges in achieving optimal HA fitting in certain scenarios. Real ear measurements, especially in cases of poor auditory performance, stagnant intervention outcomes, or notable differences in ear canal volume, can provide valuable insights for individualized and more precise HA fittings. Specifically, applying reference RECDs during HA fitting may lead to under- or over-amplification in low- and high-frequency regions, respectively. The use of individually measured RECDs becomes crucial in cases where children might be inappropriately fitted based on general reference values. Whenever feasible, incorporating individual RECD measurements during the HA fitting and selection process is essential [17,26]. Existing research also advocates for the use of fit-to-target methods and RECD measurements to optimize hearing aid output and enhance auditory outcomes [27]. In light of our study’s findings, recommending the measurement of RECDs at low frequencies for younger children and high frequencies for older children is suggested, especially when a child’s rehabilitation progress is limited.

### Limitations and future studies

There were some limitations to this study. First, the reference RECDs may vary between different HA verification equipment. This study only examined the reference values for RM500 (Version 3.24). Future studies are warranted to investigate the reference RECDs preset in different equipment. Second, large individual differences in the age group of 0 to 2 years of age were observed in our data; however, the number of participants in this age group was less than that in other age groups. Future studies should recruit more children in this age range. Third, in this study, external ear canal geometry was not measured, which may explain the difference between the measured and reference RECDs. Future studies should attempt to measure the earmold size to obtain the cross-sectional diameter of the outer ear canal and measure the probe tube length as the length of the ear canal, to further investigate the influence of the ear canal geometry.

In addition, previous studies have indicated unique characteristics of RECD measurements obtained with custom earmolds or foam tips at frequencies below 500 Hz and above 3k Hz to 4k Hz [7,28]. Therefore, future studies could analyze the RECD results obtained with a child’s custom earmold instead of foam tips to better understand its impact on HA verification. Furthermore, age was treated as a categorical variable in the linear mixed effects model. This decision was driven by the fact that age determines the reference RECD values, and across age, the reference RECDs exhibit a stepwise function rather than a smooth curve. However, treating age as categorical may introduce challenges to statistical assumptions about normality in the model and could impact the interpretation of the results.

Furthermore, it’s important to note that age-based RECD values exhibited uniformity within each age group, contributing to negligible normative RECD variability. Conversely, measured RECDs displayed significant variability. This discrepancy in variability necessitates a nuanced interpretation of statistical significance when comparing measured and normative RECDs within an age group. The observed significance may, in part, be attributed to inherent differences in variability rather than solely to substantive differences in RECD values. This aspect should be considered in future studies aiming to refine our understanding of age-related influences on RECD measurements.

Last but not the least, while the current model includes a random intercept for “ear” nested within “participant,” it may not fully capture the variability in the relationship between “ear” and “RECD” within each participant. The random intercept for “ear” allows the model to account for the correlation between the left and right ears from the same participant to some extent. Future studies could explore the potential benefits of hierarchical modeling for “ear” within the random effects side of the model for each participant to provide a more comprehensive representation of the correlation between the left and right ears.

## Conclusions

Our investigation has unveiled a statistically significant, albeit small, difference between the reference RECDs and the measured RECDs. This minor discrepancy, falling within the range of test-retest reliability, reinforces the affirmation of using a Western RECD database for Taiwanese DHH children. However, we emphasize the enduring importance of individualized HA fitting strategies, particularly in cases with stagnant intervention progress. While the use of built-in RECD reference values can provide valuable preliminary fitting guidance, especially in busy clinical settings, our study sheds light on the circumstances where caution is essential. Audiologists can efficiently allocate their time and effort by focusing on personalized RECD measurements for cases exhibiting suboptimal intervention outcomes, thereby optimizing HA gain settings effectively.
